# Supplementary material for: Hepatic Venous Pressure Gradient Predicts Further Decompensation in Cirrhosis Patients with Acute Esophageal Variceal Bleeding
Source: Diagnostics (Basel). 2023 Jul 16;13(14):2385. doi: 10.3390/diagnostics13142385 (PMC10378396; doi:10.3390/diagnostics13142385)
Supplement: Supplementary file 1 [file diagnostics-13-02385-s001.zip › diagnostics-2435184-supplementary.pdf]

diagnostics

Supplementary Materials: Hepatic Venous Pressure Gradient Predicts Further Decompensation in Cirrhosis Patients with Acute Esophageal Variceal Bleeding

Manas Vaishnav [1], Sagnik Biswas [1], Abhinav Anand [1], Piyush Pathak [1], Shekhar Swaroop [1], Arnav Aggarwal [1], Umang Arora [1], Anshuman Elhence [1], Shivanand Gamanagatti [2], Amit Goel [3], Ramesh Kumar [4] and Shalimar [1,*]

Supplementary text

Method of Hepatic venous pressure gradient assessment

HVPG was measured on the day of the AVB within 8 hours of endotherapy [36] Under strict aseptic precautions and non-invasive vital monitoring (pulse oximetry, electrocardiography, and blood pressure), access to the right internal jugular vein was achieved under direct ultrasonographic guidance using Seldinger's technique after application of a local anesthetic agent (2% Lignocaine). An 18 G needle was used to puncture the internal jugular vein. A guidewire was passed through the needle, and a 7 Fr arterial sheath was inserted over the guide wire. A Swan Ganz catheter (Edward Life Sciences USA) was passed under fluoroscopic guidance through the sheath into the right atrium and inferior vena cava and advanced into any one of the three hepatic veins (right, middle or left). The position was confirmed using a radio-opaque contrast injection after inflating the balloon of the catheter with 1.5 mL of air. A sinusoidogram was carried out to note the presence of any intrahepatic venovenous collaterals. FHVP was measured by keeping the tip of the catheter in the hepatic vein approximately 2-4 cm away from the opening of the vena cava. WHVP was measured after inflating the balloon with 1.5 ml of air and occluding the hepatic vein. The position (i.e., wedge) was confirmed by injecting 5 mL of contrast under fluoroscopy. All measurements were repeated, and a variance of up to 1 mm of Hg was considered acceptable for each pressure reading. At least three consistent paired values (FHVP and WHVP) were taken for each patient.

Overall decompensation after AVB in the entire AVB cohorts

After the initial presentation with AVB, 62 (42.8%) patients developed further decompensation, with new-onset/worsening ascites being the most common decompensating event in 38 (26.2%) patients, followed by GI bleeding in 36 (24.8%) patients. Details of overall further decompensation and comparison of the esophageal and gastric AVB cohort, as well as predictors of further decompensation, are shown in Supplementary Tables S6–S9 and Supplementary Figure S6.

Further decompensation based on time of clinical presentation

Data based on time of clinical presentation were analyzed, categorizing all decompensations within 42-days of the first presentation to be related to the index variceal bleeding, and those developing after 42-days of index presentation as further decompensation.

Decompensation within 42 days of AVB

Whole AVB cohort

Twenty-six (17.9%) patients decompensated after a median duration of 13 (2-25) days. New-onset/ worsening ascites was the most common decompensating event in 19 (13.1%) patients, followed by GI bleeding, which occurred in 10 (6.9%).(Supplementary Table S10).

Comparison of decompensation within 42 days in esophageal and gastric AVB cohort

In the esophageal and gastric AVB groups, a total of 19 (18.4%) and 7 (16.7%) patients decompensated within 42 days. The most common decompensations in the two groups were new-onset/worsening ascites in 13 (12.6%) and GI bleeding in 7 (16.7%), respectively. The details of other decompensating events are shown in Supplementary Table S10. On the multivariate model, predictors of decompensation within 42 days of AVB in the esophageal group included CTP score, serum albumin, and blood urea (Supplementary Table S11). In the gastric AVB group, there were no independent predictors of decompensation (Supplementary Table S12).

References

36. Bochnakova T. Hepatic Venous Pressure Gradient. *Clin Liver Dis*. 2021;17(3):144-148. doi:10.1002/cld.1031

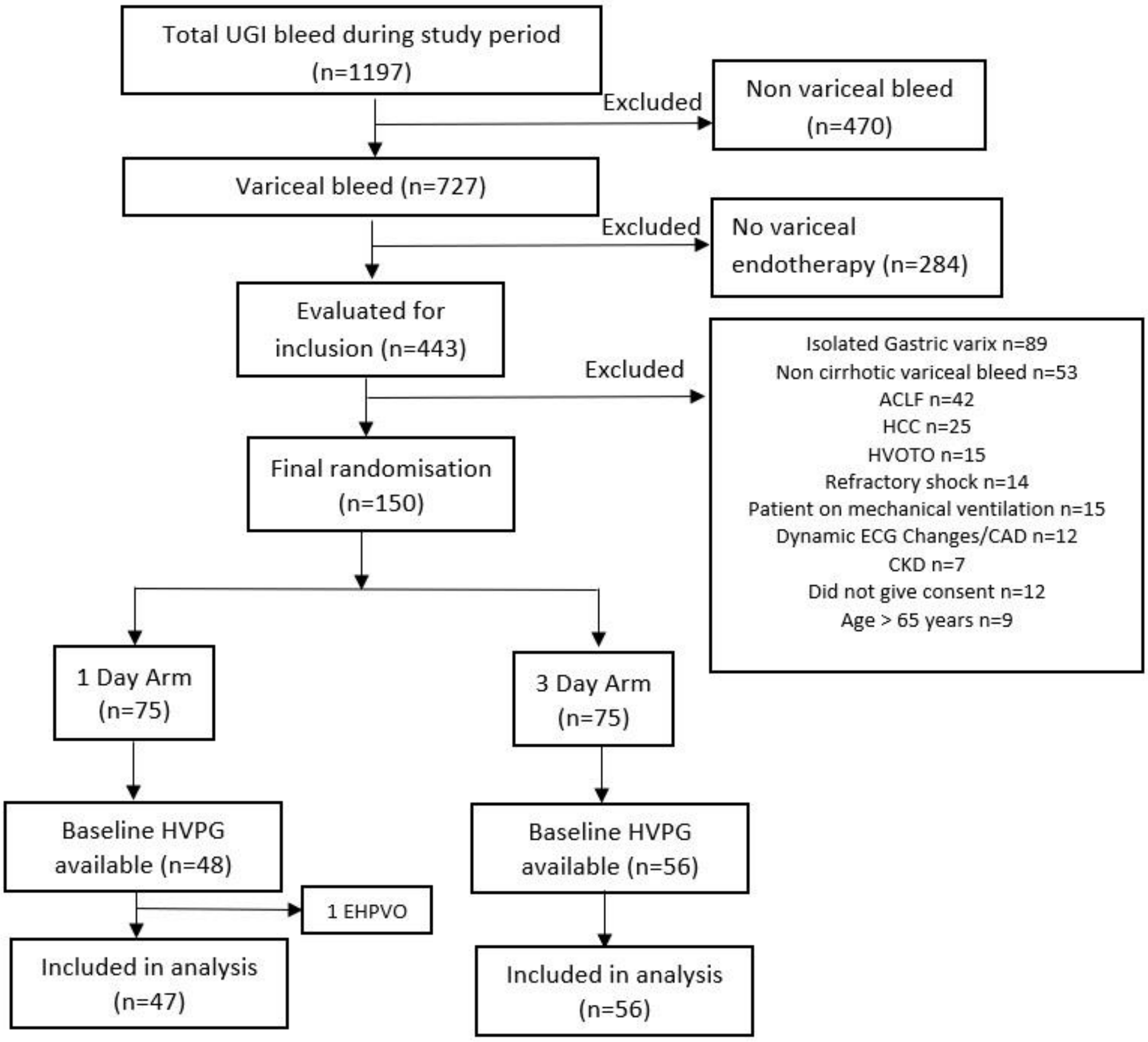

Figure S1. Consort chart for esophageal AVB (Study 1).

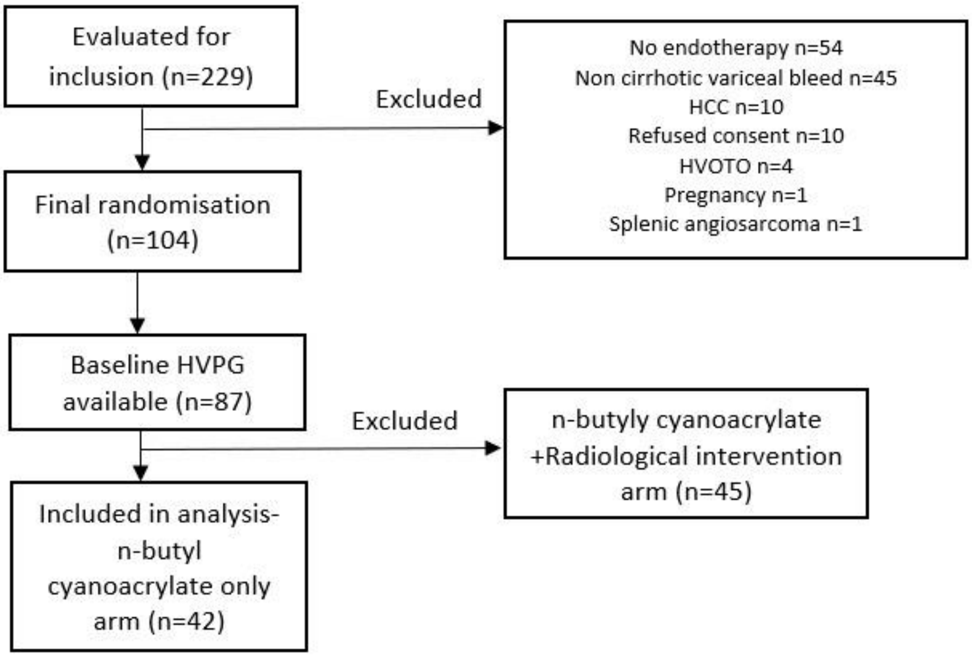

Figure S2. Consort chart for gastric AVB (Study 2).

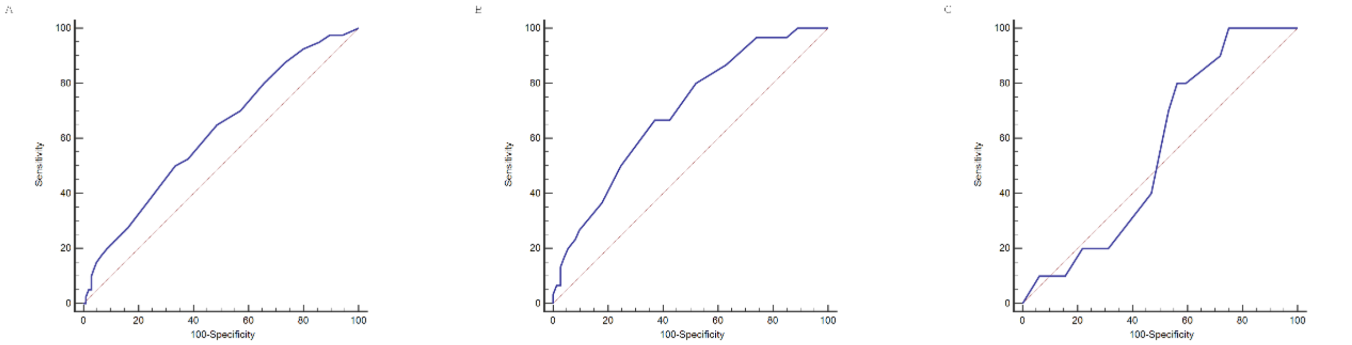

Figure S3. HVPG as a predictor of further decompensation (beyond 42 days of acute variceal bleeding), (a) whole cohort, AUROC, 0.619 (0.535-0.699), (b) esophageal acute variceal bleeding, AUROC, 0.698 (0.599-0.784), (c) gastric variceal bleeding, 0.556 (0.395-0.709).

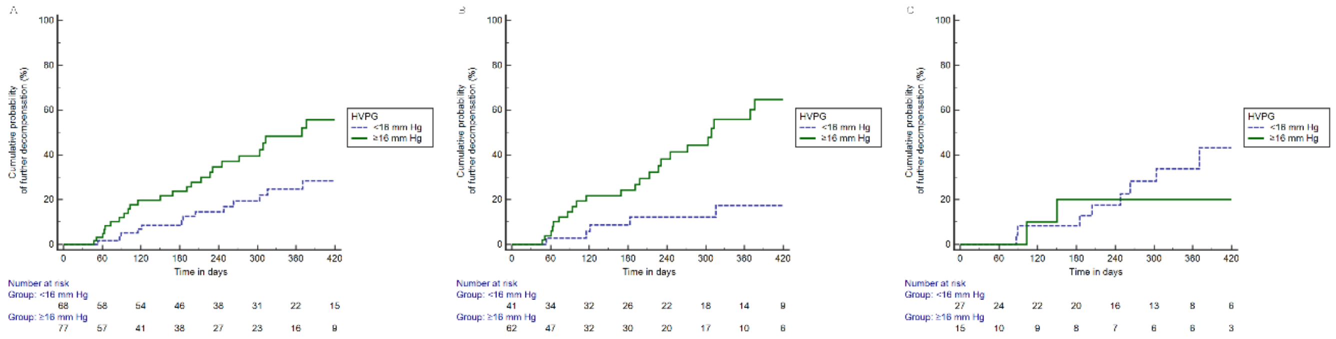

Figure S4. HVPG as a predictor of further decompensation (beyond 42 days of acute variceal bleeding), (a) whole cohort, AUROC, 0.619 (0.535-0.699), (b) esophageal acute variceal bleeding, AUROC, 0.698 (0.599-0.784), (c) gastric variceal bleeding, 0.556 (0.395-0.709). Comparing cumulative probability of further decompensation (after 42 days) in patients having HVPG <16 mm Hg and HVPG ≥ 16 mm Hg (a) whole cohort (log rank P= 0.012), (b) Esophageal AVB (log rank P= 0.002), (c) Gastric AVB (log rank P= 0.451)

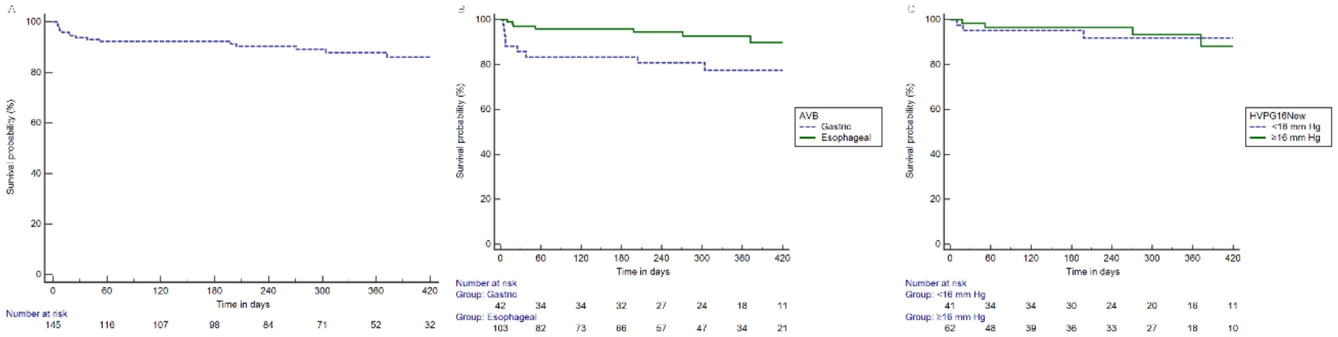

Figure S5. Kaplan Meier curve showing cumulative probablity of survival in (a) whole cohort, (b) comparing esophageal and gastric AVB group (log rank P= 0.039), (c) comparing HVPG < 16 mm Hg and ≥ 16 mm Hg in esophageal AVB group (log rank P= 0.682).

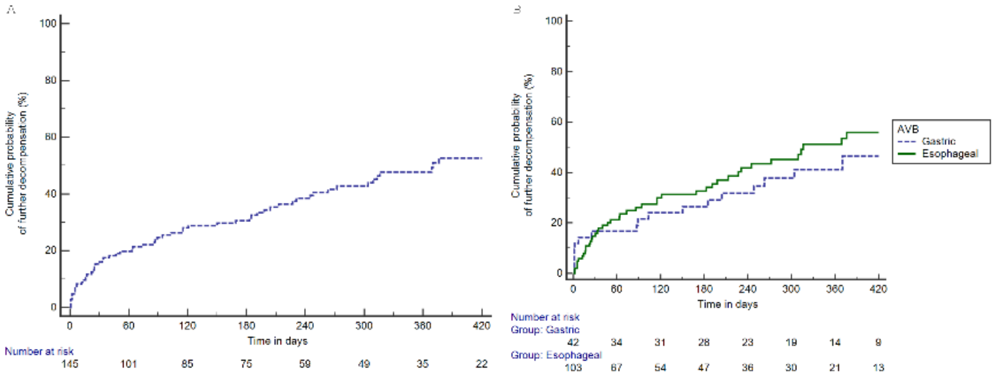

Figure S6. Cumulative probablity of further decompensation (at any time) in (a) whole cohort and (b) comparision of esohageal and gastric AVB group (log rank P = 0.415).

Table S1. Univariate and multivariate predictors of further decompensation (>42 days) in gastric AVB.

Parameters	Univariate Model		Multivariate Model-1		Multivariate Model-2 (Includes CTP score)	
	HR	P	HR	P	HR	P
Age	0.998 (0.951-1.048)	0.949				
Sex (Female)	0.239 (0.030-1.910)	0.177				
Hemoglobin (g/dL)	1.073 (0.877-1.313)	0.492				
Platelets (x103/cu.mm)	1.004 (1.000-1.007)	0.040	1.005 (1.001-1.009)	0.016	1.001 (0.997-1.004)	0.777
Creatinine (mg/dl)	1.664 (0.444-6.238)	0.450				
INR	1.500 (0.521-4.319)	0.452				
Bilirubin (mg/dl)	1.559 (1.125-2.161)	0.008	2.071 (1.080-3.971)	0.028		
Albumin (g/dL)	0.716 (0.298-1.721)	0.456	`			
Sodium (meq/L)	0.861 (0.752-0.986)	0.031	0.880 (0.766-1.012)	0.073	0.830 (0.709-0.973)	0.022
CTP Score	1.614 (1.156-2.254)	0.005			1.667 (1.121-2.480)	0.012
MELD score	1.089 (0.980-1.210)	0.112				
Ascites, yes	3.620 (0.935-14.019)	0.063	2.049 (0.430-9.775)	0.368		
Diabetes, yes	0.952 (0.202-4.494)	0.951				
HVPG mmHg	0.945 (0.818-1.092)	0.445				

Note: Multivariate model-1, all significant variables included; Multivariate model-2, includes CTP score (excludes INR, bilirubin, albumin and ascites). Abbreviations: AVB,Acute variceal bleeding; CTP, Child Turcotte Pugh; HVPG, Hepatic venous pressure gradient; INR, International normalised ratio; MELD, Model for end-stage liver disease

Table S2. Overall mortality and causes of death.

Parameters	Overall cohort (n=145) (%)	Esophageal AVB (n=103)	Gastric AVB (n=45)	P-value
Death	17 (11.7%)	8 (7.8%)	9 (20%)	0.020
GI bleed	13 (76.5%)	6 (75%)	7 (77.8%)	
Progressive liver failure	4 (23.5%)	2 (25%)	2 (22.2%)	

Table S3. Univariate and multivariate predictors of death in whole cohort of esophageal and gastric AVB.

Univariate Model			Multivariate Model-1 (All significant continuous variable)		Multivariate Mode-2 (Includes MELD)		Multivariate Model-3 (Includes CTP score)	
Parameters	HR	P	HR	P	HR	P	HR	P
Age (years)	1.072 (0.992-1.020)	0.006	1.087 (1.026-1.151)	0.004	1.089 (1.034-1.148)	0.001	1.076 (1.023-1.132)	0.005
Sex (Female)	0.414 (0.055-3.125)	0.393						
Hemoglobin (g/dL)	0.952 (0.792-1.146)	0.606						
Platelets (x10^3/cu.mm)	0.999 (0.992-1.006)	0.828						
Urea (mg/dL)	1.010 (0.999-1.021)	0.088						
Creatinine (mg/dL)	3.981 (1.357-11.674)	0.012	1.908 (0.553-6.578)	0.419			2.361 (0.727-7.666)	0.153
INR	2.181 (1.041-4.569)	0.039	2.346 (0.941-5.849)	0.067				
Total bilirubin (mg/dL)	1.160 (1.009-1.333)	0.037	1.070 (0.903-1.269)	0.433				
Albumin (g/dL)	0.637 (0.292-1.390)	0.257						
Sodium (mEq/L)	0.912 (0.824-1.009)	0.074	0.983 (0.883-1.094)	0.757			0.948 (0.858-1.046)	0.287
CTP Score	1.414 (1.073-1.862)	0.014					1.466 (1.092-1.967)	0.011
MELD score	1.143 (1.067-1.226)	<0.001			1.157 (1.062-1.260)	0.001		
Ascites, yes	10.002 (2.281-43.858)	0.002	6.822 (1.453-32.030)	0.015	6.521 (1.406-30.247)	0.017		
Diabetes, yes	0.566 (0.129-2.480)	0.450						
HVPG (mmHg)	1.019 (0.928-1.119)	0.692						

Note: Multivariate model-1, all significant variables included; multivariate model-2, includes MELD score (excludes creatinine,INR,bilirubin); Multivariate model-3, includes CTP score (excludes INR,bilirubin,albumin,ascites). Abbreviations: AVB,Acute variceal bleeding; CTP,Child Turcotte Pugh; HVPG, Hepatic venous pressure gradient; INR, International normalised ratio; MELD, Model for end-stage liver disease

Table S4. Comparison of of further decompensation (after ≥43 days) in patients with esophageal AVB receiving-1-day vs 3 days terlipressin.

Parameters	1 day terlipressin (n=47)	3 days terlipressin (n=56)	P value
Any further decompensation	16 (34%)	14 (25%)	0.314
Ascites	4 (8.5%)	12 (21.4 %)	0.071
GI bleed	12 (25.5%)	6 (10.7 %)	0.068
HE	0	5 (8.9 %)	0.061
Jaundice	3 (6.4%)	2 (3.6%)	0.658
Death	2 (4.3%)	3 (5.4%)	1.000

Table S5. Comparison of further decompensation (after ≥43 days) in patients of gastric AVB- with and without radiological intervention.

Parameters	Gastric AVB-Without intervention (n=42)	Gastric AVB- With intervention (n=45)	P value
Any further decompensation	10 (23.8%)	13 (28.9%)	0.591
Ascites	4 (9.5%)	10 (22.2%)	0.107
GI bleed	9 (21.4%)	3 (6.7%)	0.046
HE	1 (2.4%)	7 (15.6%)	0.034
Jaundice	2 (4.8%)	3 (6.7%)	0.703
Death	2 (4.8%)	5 (11.1%)	0.277

Table S6. Any further decompensation at follow-up in the overall cohort, esophageal, and gastric AVB cohorts.

Parameters	Overall cohort (n=145)	Esophageal AVB (n=103)	Esophageal AVB HVPG <16 mm Hg (n=41)	Esophageal AVB HVPG ≥16 mm Hg (n=62)	P value*	Gastric AVB (n=42)	P value**
Any further decompensation	62 (42.8%)	45 (43.7%)	11 (26.8%)	34 (54.8%)	0.005	17 (40.5%)	0.723
Median duration(days)	79 (17-216)	64 (19-220)	33 (14-183)	68 (23-228)	0.561	89 (2-226)	0.533
Type of further decompensation							
Ascites (new or worsening)	38 (26.2%)	28 (27.2%)	6 (14.6%)	22 (35.5%)	0.020	10 (23.8%)	0.675
GI Bleed	36 (24.8%)	20 (19.4%)	3 (7.3%)	17 (27.4%)	0.012	16 (38.1%)	0.018
HE	13 (9%)	8 (7.8%)	3 (7.3%)	5 (8.1%)	0.890	5 (11.9%)	0.523
Jaundice	9 (6.2%)	5 (4.9%)	2 (4.9%)	3 (4.8%)	0.993	4 (9.5%)	0.283
Death	17 (11.7%)	8 (7.8%)	3 (7.3%)	5 (8.1%)	0.890	9 (21.4%)	0.042

Abbreviations: AVB, Acute variceal bleeding; GI, Gastrointestinal; HE, Hepatic encephalopathy. Note: *comparison between Esophageal AVB < 16 mm Hg and ≥16 mmHg; **comparison between Esophageal AVB and Gastric AVB

Table S7. Univariate and multivariate predictors of further decompensation anytime in whole cohort (esophageal and gastric AVB).

Univariate Model			Multivariate Model-1 (All significant continuous variable)		Multivariate Model-2 (Includes MELD)		Multivariate Model-3 (Includes CTP score)	
Parameters	HR	P	HR	P	HR	P	HR	P
Age (years)	1.005 (0.982-1.030)	0.653						
Sex (Female)	0.819 (0.372-1.804)	0.620						
Hemoglobin (g/dL)	0.930 (0.843-1.025)	0.144						
Platelets (x10^3/cu.mm)	1.001 (0.998-1.004)	0.537						
Urea (mg/dL)	1.007 (1.001-1.013)	0.032						
Creatinine (mg/dL)	1.942 (1.042-3.617)	0.037	1.186 (0.597-2.354)	0.626			1.385 (0.728-2.636)	0.322
INR	2.196 (1.483-3.252)	<0.001	1.83 (0.748-1.870)	0.472				
Total bilirubin (mg/dL)	1.249 (1.154-1.352)	<0.001	1.193 (1.085-1.312)	<0.001				
Albumin (g/dL)	0.380 (0.238-0.609)	<0.001	0.585 (0.344-0.995)	0.048	0.551 (0.328-0.925)	0.024		
Sodium (mEq/L)	0.917 (0.866-0.971)	0.003	0.959 (0.904-1.018)	0.167			0.941 (0.888-0.998)	0.044
CTP Score	1.616 (1.392-1.876)	<0.001					1.568 (1.342-1.832)	<0.001
MELD score	1.124 (1.081-1.171)	<0.001			1.089 (1.042-1.138)	<0.001		
Ascites, yes	2.364 (1.410-3.964)	<0.001	1.477 (0.826-2.641)	0.189	1.377 (0.783-2.419)	0.266		
Diabetes, yes	0.889 (0.462-1.712)	0.725						
HVPG (mmHg)	1.050 (1.005-1.097)	0.029	1.048 (0.997-1.102)	0.066	1.030 (0.980-1.084)	0.246	1.061 (0.994-1.133)	0.075

Note: Multivariate model-1, all significant variables included(except creatinine); multivariate model-2, includes MELD score (excludes urea,creatinine, bilirubin, INR and sodium); multivariate model-3, includes CTP score (excludes INR,blirubin.albumin and ascites). Abbreviations: AVB,Acute variceal bleeding; CTP,Child Turcotte Pugh; HVPG, Hepatic venous pressure gradient; INR, International normalised ratio; MELD, Model for end-stage liver disease

Table S8. Univariate and multivariate predictors of further decompensation at any time in esophageal AVB.

Univariate Model			Multivariate Model-1 (All significant continuous variable)		Multivariate Model-2 (Includes MELD)		Multivariate Model-3 (Includes CTP score)	
Parameters	HR	P	HR	P	HR	P	HR	P
Age (years)	0.999 (0.967-1.032)	0.972						
Sex (Female)	2.182 (0.918-5.281)	0.070	2.674 (1.036-6.900)	0.042	2.291 (0.896-5.854)	0.083	2.191 (0.877-5.478)	0.093
Hemoglobin (g/dL)	0.889 (0.781-1.012)	0.076	0.934 (0.758-1.151)	0.520	1.009 (0.844-1.207)	0.922	0.966 (0.819-1.140)	0.685
Platelets ($x10^3$/cu.mm)	0.999 (0.993-1.004)	0.629						
Urea (mg/dL)	1.010 (1.002-1.018)	0.015	1.010 (1.001-1.020)	0.037			1.015 (1.005-1.024)	0.003
Creatinine (mg/dL)	1.994 (0.862-4.614)	0.107						
INR	2.394 (1.518-3.776)	<0.001	0.826 (0.453-1.504)	0.531				
Total bilirubin (mg/dL)	1.291 (1.155-1.443)	<0.001	1.382 (1.177-1.622)	<0.001				
Albumin (g/dL)	0.235 (0.126-0.438)	<0.001	0.454 (0.193-1.068)	0.070	0.354 (0.156-0.803)	0.013		
Sodium (mEq/L)	0.947 (0.878-1.022)	0.162						
CTP Score	1.860 (1.517-2.279)	<0.001					1.902 (1.533-2.360)	<0.001

MELD score	1.125 (1.074-1.179)	<0.001			1.089 (1.035-1.145)	0.001		
Ascites, yes	2.109 (1.155-3.850)	0.015	1.414 (0.721-2.775)	0.314	1.154 (0.598-2.226)	0.669		
Diabetes, yes	1.060 (0.507-2.217)	0.877						
HVPG (mmHg)	1.077 (1.019-1.139)	0.009	1.081 (1.013-1.153)	0.019	1.051 (0.984-1.122)	0.140	1.073 (1.005-1.145)	0.034

Note: Multivariate model-1, all significant variables included; Multivariate model-2, includes MELD score (excludes urea,INR and bilirubin); Multivariate model-3, includes CTP score (excludes INR,bilirubin,albumin and ascites). Abbreviations: AVB,Acute variceal bleeding; CTP,Child Turcotte Pugh; HVPG, Hepatic venous pressure gradient; INR, International normalised ratio; MELD, Model for end-stage liver disease.

Table S9. Univariate and multivariate predictors of further decompensation at anytime in patients with gastric AVB.

	Univariate Model		Multivariate Model (All significant continuous variable)		Multivariate Model (Includes MELD)		Multivariate Model (Includes CTP score)	
Parameters	HR	P	HR	P	HR	P	HR	P
Age (Years)	1.019 (0.980-1.060)	0.345						
Sex (Female)	0.166 (0.022-1.265)	0.083	0.271 (0.033-2.222)	0.224	1.092 (0.991-1.203)	0.075	0.274 (0.034-2.222)	0.225
Hemoglobin (g/dL)	1.014 (0.862-1.192)	0.866						
Platelets (x10^3/cu.mm)	1.002 (0.999-1.005)	0.187						
Urea (mg/dl)	1.005 (0.994-1.016)	0.356						
Creatinine (mg/dL)	2.169 (0.802-5.868)	0.127						
INR	1.889 (0.911-3.914)	0.087	01.333 (0.577-3.078)	0.500				
Total bilirubin (mg/dL)	1.245 (1.090-1.422)	0.001	1.162 (1.005-1.343)	0.042				
Albumin (g/dL)	0.750 (0.387-1.453)	0.394						
Sodium (mEq/L)	0.860 (0.775-0.954)	0.004	0.895 (0.800-1.002)	0.053			0.856 (0.766-0.956)	0.006
CTP Score	1.418 (1.090-1.846)	0.009					1.384 (1.054-1.816)	0.019
MELD score	1.141 (1.050-1.239)	0.002			1.092 (0.991-1.203)	0.075		
Ascites, yes	3.328 (1.169-9.473)	0.024	1.588 (0.493-5.118)	0.439	1.609 (0.467-5.545)	0.451		
Diabetes, yes	0.550 (0.125-2.408)	0.427						
HVPG (mmHg)	0.995 (0.907-1.091)	0.912						

Note: Multivariate model-1, all significant variables included; multivariate model-2, includes MELD score (excludes INR,bilirubin); multivariate model-3, includes CTP score (excludes INR,bilirubin,albumin,ascites). Abbreviations: AVB,Acute variceal bleeding; CTP,Child Turcotte Pugh; HVPG, Hepatic venous pressure gradient; INR, International normalised ratio; MELD, Model for end-stage liver disease.

Table S10. Further decompensation within 42 days in the overall cohort, esophageal, and gastric AVB cohorts.

Parameters	Overall cohort (n=145)	Esophageal AVB (n=103)	Esophageal AVB HVPG <16 mm Hg (n=41)	Esophageal AVB HVPG ≥16 mm Hg (n=62)	P value*	Gastric AVB (n=45)	P value**
Any further decompensation	26 (17.9%)	19 (18.4%)	6 (14.6%)	13 (21.0%)	0.417	7(16.7%)	0.800
Median duration (Days)	13 (2-25)	17 (5-26)	22 (2-40)	16 (5-26)	0.895	1 (1-7)	0.007
Types of further decompensation							
New onset Ascites/Worsening	19 (13.1%)	13 (12.6%)	4 (9.8%)	9 (14.5%)	0.476	6 (14.3%)	0.788
GI Bleed	10 (6.9%)	3 (2.9%)	0	3 (4.8%)	0.274	7 (16.7%)	0.007
HE	8 (5.5%)	4 (3.9%)	2 (4.9%)	2 (3.2%)	1.000	4 (9.5%)	0.177
Jaundice	2 (1.4%)	0	0	0	NA	2 (4.8%)	0.082
Death	10 (6.9%)	3 (2.9%)	2 (4.9%)	1 (1.6%)	0.562	7 (16.7%)	0.007

Note: Abbreviations: AVB, Acute variceal bleeding; GI, Gastrointestinal;HE,Hepatic encephalopathy; *comparison between Esophageal AVB < 16 mm Hg and ≥16 mmHg; **comparison between Esophageal AVB and Gastric AVB.

Table S11. Univariate and multivariate predictors of further decompensation within 42 days in patients with esophageal AVB.

Univariate Model			Multivariate Model-1 (All significant continuous variable)		Multivariate Model-2 (Includes MELD)		Multivariate Model-3 (Includes CTP score)	
Parameters	HR	P	HR	P	HR	P	HR	P
Age (years)	1.026 (0.973-1.081)	0.339						
Sex (Female)	2.973 (0.985-8.976)	0.053	0.384 (.0118-1.245)	0.111	0.464 (0.144-1.494)	0.198	0.459 (0.146-1.444)	0.183
Hemoglobin (g/dL)	0.900 (0.748-1.083)	0.266						
Platelets (x10^3/cu.mm)	0.998 (0.988-1.007)	0.620						
Urea (mg/dL)	1.019 (1.008-1.029)	0.001	1.018 (1.006-1031)	0.005			1.018 (1.006-1.030)	0.003
Creatinine (mg/dL)	1.024 (0.280-3.753)	0.971						
INR	1.725 (0.854-3.485)	0.129						
Total bilirubin (mg/dL)	1.258 (1.107-1.430)	<0.001	1.232 (1.066-1.424)	0.005				
Albumin (g/dL)	0.193 (0.071-0.521)	0.001	0.479 (0.146-1.567)	0.224	0.272 (0.091-0.814)	0.020		
Sodium (mEq/L)	0.925 (0.846-1.012)	0.088	0.960 (0861-1.071)	0.465				
CTP Score	1.728 (1.318-2.266)	<0.001					1.613 (1.215-2.142)	0.001
MELD score	1.085 (1.015-1.160)	<0.016			1.044 (0.974-1.119)	0.224		
Ascites, yes	2.198 (0.865-5.589)	0.098	1.393 (0.507-3.832)	0.520	1.216 (0.444-3.334)	0.703		
Diabetes, yes	1.160 (0.385-3.495)	0.792						
HVPG (mmHg)	0.971 (0.880-1.073)	0.566						

Note: Multivariate model-1, all significant variables included; multivariate model-2, includes MELD score(excludes INR,bilirubin and urea); multivariate model-3, includes CTP score(excludes bilirubin,albumin,ascites). Abbreviations: AVB,Acute variceal bleeding; CTP,Child Turcotte Pugh; HVPG, Hepatic venous pressure gradient; INR, International normalised ratio; MELD, Model for end-stage liver disease.

Table S12. Univariate and multivariate predictors of further decompensation within 42 days in patients with gastric AVB.

Univariate Model			Multivariate Model (All significant variable)	
Parameters	HR	P	HR	P
Age (years)	1.059 (0.984-1.139)	0.127		
Sex (Female)	0.035 (0.000-98.503)	0.408		
Hemoglobin (g/dL)	0.914 (0.688-1.215)	0.537		
Platelets (x10³/cu.mm)	0.999 (0.992-1.007)	0.858		
Urea (mg/dL)	0.997 (0.978-1.016)	0.754		
Creatinine (mg/dL)	3.184 (0.690-14.697)	0.138		
INR	2.460 (0.861-7.031)	0.093	2.266 (1.11-7.751)	0.136
Total bilirubin (mg/dL)	1.163 (0.971-1.393)	0.100		
Albumin (g/dL)	0.800 (0.292-2.195)	0.665		
Sodium (mEq/L)	0.859 (0.732-1.008)	0.062	0.873 (0.743-1.026)	0.087
CTP Score	1.163 (0.750-1.805)	0.500		
MELD score	1.251 (1.062-1.472)	0.007		
Ascites, yes	2.923 (0.567-15.081)	0.200		
Diabetes, yes	0.037 (0.000-164.270)	0.441		
HVPG (mmHg)	1.050 (0.935-1.178)	0.413		

Note: Multivariate model- all significant variables included. Abbreviations: AVB, Acute variceal bleeding; CTP, Child Turcotte Pugh; HVPG, Hepatic venous pressure gradient; INR, International normalised ratio; MELD, Model for end-stage liver disease.
